# Radiomics for Precision Diagnosis of FAI: How Close Are We to Clinical Translation? A Multi-Center Validation of a Single-Center Trained Model

**DOI:** 10.3390/jcm14124042

**Published:** 2025-06-07

**Authors:** Eros Montin, Srikar Namireddy, Hariharan Subbiah Ponniah, Kartik Logishetty, Iman Khodarahmi, Sion Glyn-Jones, Riccardo Lattanzi

**Affiliations:** 1 Center for Advanced Imaging Innovation and Research (CAI^2^R), New York University Grossman School of Medicine, New York, NY 10016, USA; riccardo.lattanzi@nyulangone.org; 2 Bernard and Irene Schwartz Center for Biomedical Imaging, New York University Grossman School of Medicine, New York, NY 10016, USA; 3 MSk Lab, Imperial College London, London SW7 2AZ, UK; srikar.namireddy21@imperial.ac.uk (S.N.); k.logishetty@imperial.ac.uk (K.L.); 4 Department of Targeted Intervention, Division of Surgery & Interventional Science, University College London, London WC1E 6BT, UK; hariharan.subbiah-ponniah18@imperial.ac.uk; 5 Nuffield Department of Orthopaedics Rheumatology, and Musculoskeletal Sciences, University of Oxford, Oxford OX1 2JD, UK; sion.glyn-jones@ndorms.ox.ac.uk; 6 Radiology Department, New York University Grossman School of Medicine, New York, NY 10016, USA; iman.khodarahmi@nyulangone.org

**Keywords:** femoroacetabular impingement, radiomics, machine learning, personalized medicine

## Abstract

**Background:** Femoroacetabular impingement (FAI) is a complex hip disorder characterized by abnormal contact between the femoral head and acetabulum, often leading to joint damage, chronic pain, and early-onset osteoarthritis. Despite MRI being the imaging modality of choice, diagnosis remains challenging due to subjective interpretation, lack of standardized imaging criteria, and difficulty differentiating symptomatic from asymptomatic cases. This study aimed to develop and externally validate radiomics-based machine learning (ML) models capable of classifying healthy, asymptomatic, and symptomatic FAI cases with high diagnostic accuracy and generalizability. **Methods:** A total of 82 hip MRI datasets (31 symptomatic, 31 asymptomatic, 20 healthy) from a single center were used for training and cross-validation. Radiomic features were extracted from four segmented anatomical regions (femur, acetabulum, gluteus medius, gluteus maximus). A four-step feature selection pipeline was implemented, followed by training 16 ML classifiers. External validation was conducted on a separate multi-center cohort of 185 symptomatic FAI cases acquired with heterogeneous MRI protocols. **Results:** The best-performing models achieved a cross-validation accuracy of up to 90.9% in distinguishing among healthy, asymptomatic, and symptomatic hips. External validation on the independent multi-center cohort demonstrated 100% accuracy in identifying symptomatic FAI cases. Since this metric reflects performance on symptomatic cases only, it should be interpreted as a detection rate (true positive rate) rather than overall multi-class accuracy. Gini index-based feature selection consistently outperformed F-statistic-based methods across all the models. **Conclusions:** This is the first study to systematically integrate radiomics and multiple ML models for FAI classification for these three phenotypes, trained on a single-center dataset and externally validated on multi-institutional MRI data. The demonstrated robustness and generalizability of radiomic features support their use in clinical workflows and future large-scale studies targeting standardized, data-driven FAI diagnosis.

## 1. Introduction

Femoroacetabular impingement (FAI) is a hip disorder characterized by abnormal contact between the femoral head and acetabulum, often resulting in cartilage damage, labral tears, chronic pain, and reduced quality of life [1]. If FAI remains untreated, it may progress to osteoarthritis, highlighting the need for early and precise diagnosis. However, diagnosing FAI is fraught with difficulties due to the overlapping symptoms with other hip disorders, variability in imaging-based criteria, and inconsistencies in the reliability of physical examinations [1]. Although plain radiography remains the cornerstone of the initial investigation, magnetic resonance imaging (MRI), is the gold standard modality for assessing FAI and planning surgical intervention (Lisbon Agreement 3 [2]). MRI facilitates evaluation of hip joint morphology, injury to the labrum and/or articular cartilage, and extra-articular causes of pain. However, conventional MRI suffers from inter-user variability and lacks the quantitative metrics necessary to reliably distinguish between symptomatic and asymptomatic cases [3]. Furthermore, the lack of universally accepted imaging criteria for the diagnosis of FAI complicates clinical decision-making and limits diagnostic reproducibility [1]. This challenge is exacerbated by the dynamic and three-dimensional nature of FAI and its variability with respect to the imaging protocols. Delayed gadolinium-enhanced magnetic resonance imaging of cartilage (dGEMRIC), as well as T1ρ and T2* MRI techniques—developed to quantify biochemical changes in cartilage [2]—have had only a moderate correlation with arthroscopic findings, and no correlation with symptom state or surgical outcome [4,5]. FAI is a dynamic bony pathomorphology with region-specific labral and articular cartilage injury, so MRI-based cartilage mapping alone may be an insufficient reflection of the disease process. To address these limitations, we propose utilizing radiomics to extract a broader array of quantitative features from hip MRI, capturing shape and textural characteristics that may be imperceptible to human observers [6,7]. This includes not only traditional joint structures like the femur and acetabulum, but also periarticular soft tissues such as the gluteus medius and gluteus maximus. These muscles are integral to hip stabilization and gait mechanics and may undergo adaptive remodeling in response to joint pathology.

When combined with ML, radiomic features can be used to develop predictive models capable of classifying disease states, estimating prognosis, and even predicting therapeutic responses [7,8,9,10]. Although radiomics has already demonstrated transformative potential in oncology, its application to FAI and musculoskeletal disorders remains in the early stages [11,12,13].

Nonetheless, preliminary evidence suggests that radiomic analysis of musculoskeletal tissues can uncover meaningful patterns associated with function and symptoms. For instance, patients with unilateral FAI have been shown to exhibit asymmetries in gluteal muscle cross-sectional area that correlate with preoperative pain severity and altered load distribution [14].

Recent studies have yielded promising results, with MRI-based radiomic models achieving 100% accuracy in distinguishing between healthy individuals and those affected by FAI [15] and surpassing 97% accuracy in differentiating symptomatic hips from asymptomatic contralateral hips in unilateral FAI cases [16,17]. However, these studies are constrained by reliance on data augmentation [18] of small datasets, involving patients from a single center using the same MRI protocol, which were analyzed with a single ML model. Building on this previous work, this study aims to develop and validate a radiomic-driven ML model to improve the precision and reliability of noninvasive FAI diagnosis. Through a systematic evaluation of radiomic features with various ML models on a large multi-center cohort of data, this study aims to establish a robust and standardized method to classify healthy, symptomatic, and asymptomatic hip joints, ultimately advancing the precision and reproducibility of FAI diagnosis. This is the first study to systematically evaluate a broad range of machine learning models in combination with radiomic features for FAI classification, trained entirely on single-center data and externally validated across a large multi-institutional cohort. By testing the generalizability of radiomic signatures across variable imaging protocols, scanner vendors, and acquisition settings, this work establishes a reproducible foundation for clinical translation and supports the development of standardized, data-driven FAI diagnostics.

## 2. Materials and Methods

### 2.1. MRI Data Acquisition

**Figure 1:**
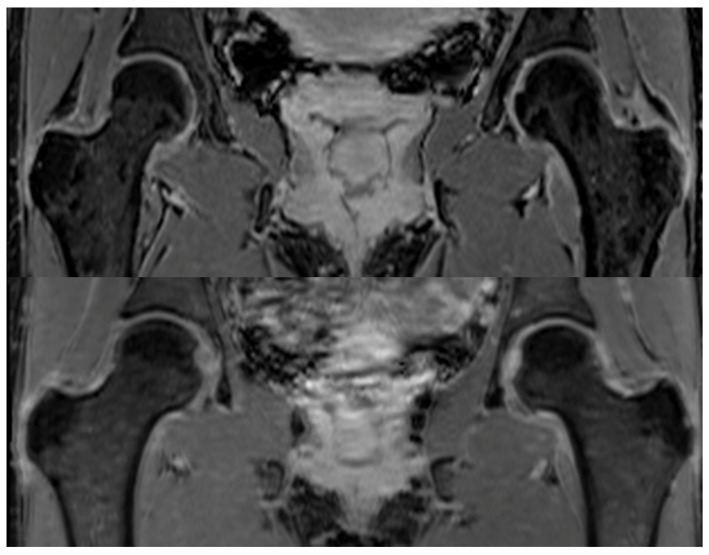
Two examples of DIXON MRI sequences highlighting the left and right femurs and the acetabulum of a patient (**top**) and a healthy subject (**bottom**). Both images were acquired using an axial dual-echo T1-weighted 3D FLASH sequence with Dixon fat–water separation, enabling detailed visualization of hip joint structures. Although the image contrast appears visually similar between the two cases, the symptomatic patient exhibits a subtle but clinically relevant narrowing of the space between the femoral head and acetabulum. These morphological changes are difficult to appreciate in isolated 2D views, and this challenge underlies the motivation for the current study, which leverages radiomic features to detect and quantify such structural differences objectively.

MRI data was obtained from three cohorts (Table 1). Cohorts 1 and 2: These included bilateral hip MRI scans from a total of 41 subjects imaged at a single center. Ethical approval for the study protocol of Cohorts 1 and 2 was obtained from the Institutional Review Board (IRB) before data collection and analysis commenced. Cohort 1 comprised 31 patients with confirmed unilateral FAI (22 females, mean age: 36 ± 8 years), of which 20 scans included contrast-enhanced sequences. All subjects in Cohort 1 were surgically confirmed to exhibit mixed-type FAI (cam + pincer). Cohort 2 consisted of 10 healthy volunteers (5 females, mean age: 32 ± 6 years), scanned without contrast. All MRI exams for these two cohorts were acquired using a consistent imaging protocol: an axial dual-echo T1-weighted three-dimensional (3D) FLASH sequence with Dixon fat–water separation. Acquisition parameters were as follows: repetition time (TR) = 10 ms, echo times (TEs) = 2.4 ms and 3.7 ms, field of view (FOV) = 32 cm, acquisition matrix = 320 × 320, and slice thickness = 1 mm (Figure 1).

Cohort 3: This external validation dataset included 185 symptomatic FAI cases sourced from seven different institutions as part of the FAIT trial [19]. Ethical approval for the trial protocol of Cohort 3 was approved by the Health Research Authority, National Research Ethics Services Committee South Central–Berkshire (REC reference: 13/SC/0154) and by local research and development departments at each participating site. All patients had unilateral symptomatic FAI, confirmed by clinical and radiographic assessment.

Detailed morphological phenotyping was available for Cohort 3 as part of the FAIT trial. Among the 185 patients, 140 were classified as cam-type, 28 as cam with associated dysplasia, 2 as isolated pincer, 14 as cam + pincer, and 1 as isolated dysplasia.

**Figure 2:**
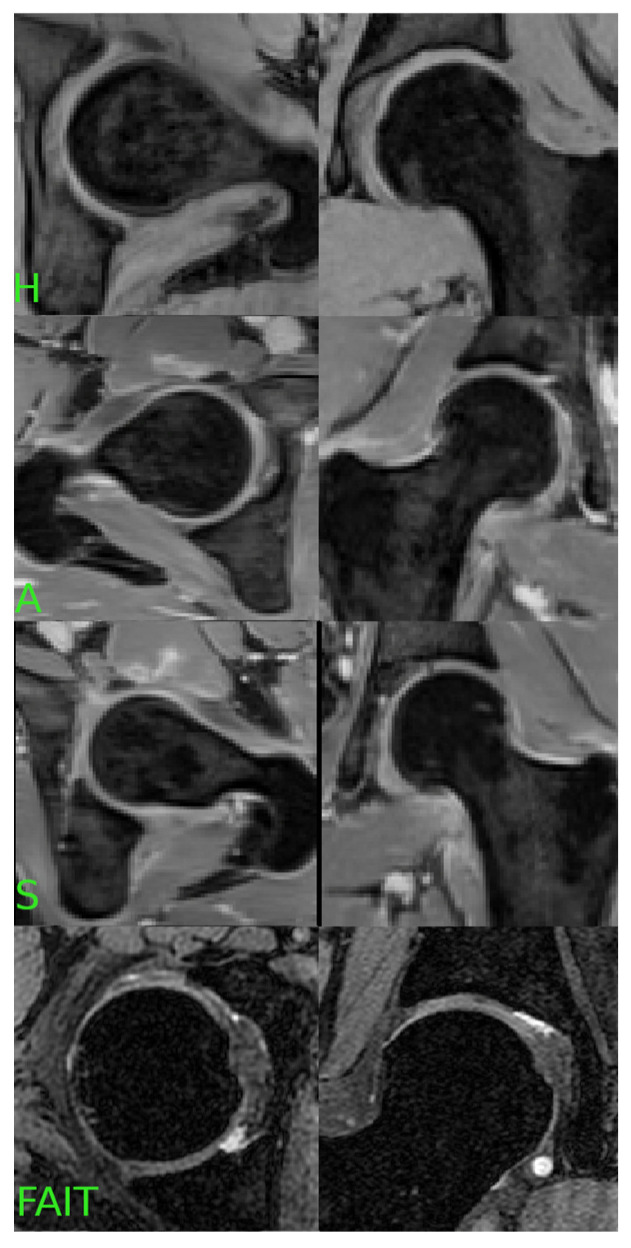
The dataset was labeled according to the presence of hip pain: “H” for healthy hips, “A” for asymptomatic FAI, “S” for symptomatic FAI and the Cohort 3 (FAIT) dataset. This labeling scheme enabled the classification of hip conditions based on radiomic features extracted from the segmented ROIs.

Unlike the training data, the images in Cohort 3 were acquired across a heterogeneous range of 1.5T and 3T MRI scanners using different vendor platforms and site-specific implementations of water-only Dixon sequences. Imaging parameters varied slightly across centers and were not standardized, which reflects real-world variability in clinical imaging practices. This cohort was used exclusively for external testing to evaluate the generalizability of the radiomic features and machine learning models. The deliberate use of heterogeneous imaging in the external validation cohort was intended to simulate deployment conditions and assess the resilience of the trained models to scanner and protocol variation. An example of the data in the three cohorts can be found in Figure 2.

### 2.2. Region of Interest Segmetation

All MRI datasets across the three cohorts, including morphological scans when available, were segmented using TotalSegmentator v2.2.1 [20], after being resampled to a uniform resolution of 1 mm isotropic using a B-spline interpolator from the ITK library [21,22,23]. The segmentation targeted four anatomically and functionally relevant regions: the femur, acetabulum, gluteus maximus, and gluteus medius. While previous radiomics studies on FAI have focused primarily on osseous structures [15], we intentionally extended our analysis to include the gluteal muscles. This decision was motivated by evidence that FAI alters neuromuscular activation and loading patterns, leading to compensatory hypertrophy or atrophy in periarticular muscle groups [14]. In particular, asymmetries in gluteal muscle cross-sectional area have been shown to correlate with preoperative pain and altered biomechanics in FAI patients [17]. By extracting radiomic features from these soft-tissue structures, we aimed to capture complementary pathophysiological information that may enhance discrimination between symptomatic and asymptomatic cases.

**Figure 3:**
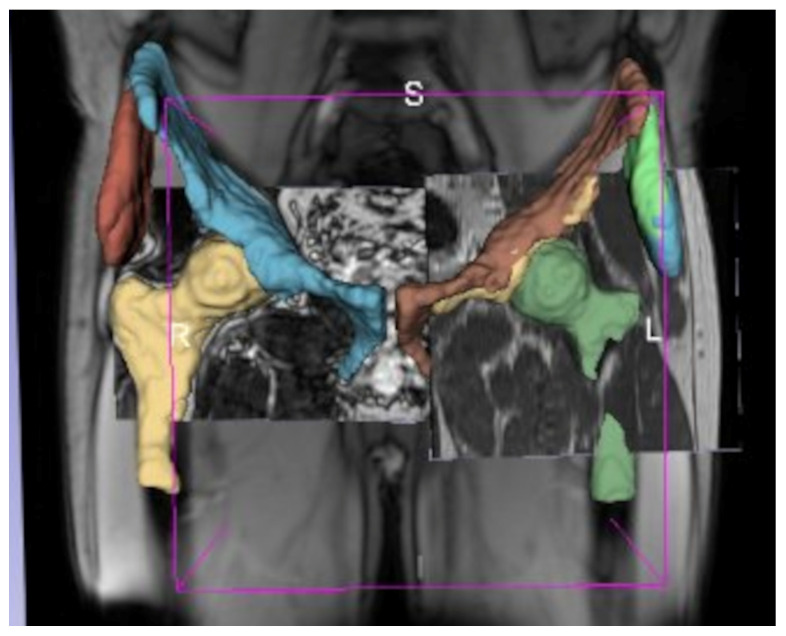
Illustration of the STAPLE algorithm combining three distinct segmentations with varying fields of view to generate a consensus segmentation of four ROIs. By probabilistically weighting each input segmentation, STAPLE improves accuracy and robustness of the final segmentation output. S, superior; L, left; R, right.

For each subject, the segmented structures were merged into a single composite segmentation using the Simultaneous Truth and Performance Level Estimation (STAPLE) algorithm [24] to increase robustness and minimize noise from automated outputs (Figure 3). Final segmentations were visually reviewed and quality-checked by a musculoskeletal radiologist (I.K., >10 years of experience).

### 2.3. Dataset Preparation

In Cohorts 1 and 2, water-only images and the associated segmentations were separated into left and right hip regions for each subject. For patients in Cohort 1 with unilateral symptoms, the clinically affected hip was labeled as “S” (symptomatic), while the contralateral hip—confirmed to be pain-free and lacking morphological features of impingement—was labeled as “A” (asymptomatic). Importantly, we did not assume bilateral symmetry of FAI; rather, each hip was independently evaluated based on clinical records and imaging review by a musculoskeletal radiologist.

For Cohort 2, which included healthy volunteers, both hips were labeled as “H” (healthy) based on the absence of symptoms and imaging findings suggestive of FAI.

For Cohort 3, only the symptomatic side was available per subject. All 185 cases were thus labeled as “S”, representing confirmed symptomatic FAI based on the clinical criteria defined in the FAIT study [19].

Using this schema, we constructed a training dataset of 82 hips from Cohorts 1 and 2 (20 H, 31 A, 31 S) and an external validation dataset of 185 symptomatic hips (S) from Cohort 3.

### 2.4. Feature Extraction

Radiomic features were systematically extracted from all segmented regions using PyRadiomics [25] though ad hoc package, capturing quantitative information on both bone and soft tissue structure. The extracted features encompassed first-order intensity statistics, shape descriptors, and multiple families of texture features derived from gray-level matrices including the following:

- Gray Level Co-occurrence Matrix (GLCM).
- Gray Level Run Length Matrix (GLRLM).
- Gray Level Size Zone Matrix (GLSZM).
- Neighboring Gray Tone Difference Matrix (NGTDM).
- Gray Level Dependence Matrix (GLDM).

To enhance feature diversity and multiscale analysis, we applied a series of image transformations to the 3D water-only Dixon images prior to feature extraction. These included wavelet decompositions, Laplacian of Gaussian (LoG) filters, and nonlinear intensity transforms (e.g., logarithmic, exponential, square root), among others. These transformations emphasized edges, frequency components, and local gradients relevant to structural heterogeneity.

Notably, by including the gluteus medius and maximus in the analysis, we enabled the capture of soft-tissue texture patterns that may reflect neuromuscular compensation or chronic adaptation associated with FAI. For example, texture asymmetry or homogeneity in these muscle groups could indicate load redistribution due to joint dysfunction.

Texture features were calculated at multiple scales using radii of 1, 2, and 4 voxels to account for spatial context. Intensities were discretized into 16, 32, 64, and 128 gray-level bins, with histogram bounds determined from each image’s minimum and maximum intensity values. In total, 74,272 features were generated per patient across all anatomical regions and transformations, providing a rich multiscale representation of structural and textural characteristics.

### 2.5. Feature Selection

**Figure 4:**
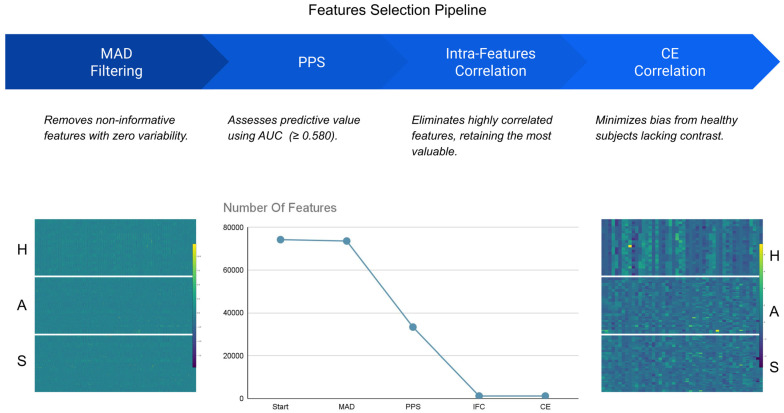
The multi-step feature selection pipeline refined the high-dimensional radiomic feature space, retaining only those features with robust prognostic significance and minimal redundancy.

Given the high-dimensional nature of the radiomic feature space (74,272 features in total, including 11,818 per region of interest), a four-step selection pipeline was applied to identify the most informative and non-redundant predictors (Figure 4) [26]. All feature selection was performed exclusively on the training dataset (Cohorts 1 and 2), ensuring that no information from the external validation cohort (Cohort 3) influenced feature ranking or model training.

Features were first normalized using Z-score transformation to ensure consistent scaling across intensity and texture domains [27]. The four filtering steps were as follows:

1. Median Absolute Deviation (MAD) Filtering—Features with zero MAD were removed as non-informative, reducing the set to 73,634.
2. Prognostic Performance Score (PPS)—Each feature was evaluated independently using a Random Forest Classifier (RFC), retaining features with an area under the ROC curve (AUC) ≥ 0.580, narrowing the set to 33,358.
3. Intra-Feature Correlation Elimination—Highly correlated feature pairs (Pearson r ≥ 0.8) were filtered by keeping only the more prognostic feature, resulting in 1186 retained features.
4. Contrast Enhancement Bias Removal—Features highly correlated (r ≥ 0.75) with a binary contrast-enhancement array (indicating contrast presence/absence) were excluded to prevent model bias, leaving 1183 final features.

For final optimization, two complementary ranking strategies were applied:

- F one-way ANOVA (SelectKBest, Scikit-learn) for global statistical group separation.
- Gini Index for model-based feature importance, capturing interactions relevant to tree-based classifiers [28].

We chose not to apply dimensionality reduction methods such as PCA [29], UMAP [30,31], or t-SNE [32] to preserve feature interpretability, which is essential for understanding anatomical contributions to model decisions and enabling clinical translation.

Feature sets were evaluated using stratified resampling (200 iterations), and top-performing subsets (1 to 50 features) were selected for training the machine learning classifiers.

### 2.6. Machine Learning

To evaluate the diagnostic potential of the radiomic features selected during the feature selection phase, we trained and compared 16 machine learning classifiers, encompassing a diverse range of algorithms, from tree-based models to linear, probabilistic, and ensemble learners. This diversity was selected to ensure a comprehensive benchmark across modeling paradigms relevant to radiomics and clinical imaging.

Each model was trained to classify hip status into one of three categories, healthy (H), asymptomatic FAI (A), and symptomatic FAI (S), using only data from Cohorts 1 and 2 for model development. The classifiers included the following:

- Tree-Based Models: Random Forest (RFC), Decision Tree (DTC), and Extra Trees (ETC) are widely used in radiomics due to their capacity to handle high-dimensional, non-linear feature interactions while maintaining interpretability.
- Boosting Models: Gradient Boosting (GBC), AdaBoost (ABC), XGBoost (XGB), LightGBM (LGBM), and CatBoost (CB) were included for their iterative correction mechanisms, which improve predictive accuracy and have shown success in radiogenomic and musculoskeletal imaging tasks.
- Linear/Probabilistic Models: Logistic Regression (LR), Quadratic Discriminant Analysis (QDA), and Gaussian Naïve Bayes (GNB) offer interpretability and statistical grounding, providing baseline comparators for complex ensembles.
- Distance and Ensemble Models: Support Vector Classifier (SVC), K-Nearest Neighbors (KNN), Bagging Classifier (BC), Stacking Classifier (SC), and Voting Classifier (VC) were selected to assess model robustness and performance under feature variability, as these methods are frequently used in imaging classification pipelines.

To prevent model bias due to class imbalance, we applied the Synthetic Minority Oversampling Technique (SMOTE) [33] to augment underrepresented class (H) during training. SMOTE generates synthetic samples by interpolating between minority class observations in feature space, effectively balancing class distribution.

All models were trained on Z-score normalized feature values using four-fold cross-validation (75/25 train-test split) to estimate generalization on unseen data. For each of the two feature selection methods (Gini and F-statistic), we evaluated subsets ranging from 1 to 50 top-ranked features, resulting in 1568 unique mode–-feature set combinations (16 classifiers × 50 subsets × 2 FS strategies).

For external evaluation, the best-performing model from each configuration (as determined by cross-validation on Cohorts 1 and 2) was applied to the entire multi-center Cohort 3, which consisted exclusively of symptomatic FAI cases. This allowed us to test the real-world diagnostic performance and generalizability of single-center–rained models to diverse MRI sources without fine-tuning.

### 2.7. Statistical Comparison

The classification performance disparity among ML models trained with features selected through two distinct techniques (Gini index and F one-way ANOVA) was assessed as follows: The mean test accuracy variations between the two FS methods were computed for each model. The statistical significance of each difference was evaluated using paired tests. Initially, the normality of the differences was examined via the Shapiro–Wilk test. For models in which normality was confirmed (*p* > 0.05), a paired t-test was employed. Conversely, for models in which normality was not observed (*p* ≤ 0.05), the Wilcoxon Signed-Rank test served as a nonparametric alternative.

## 3. Results

**Figure 5:**
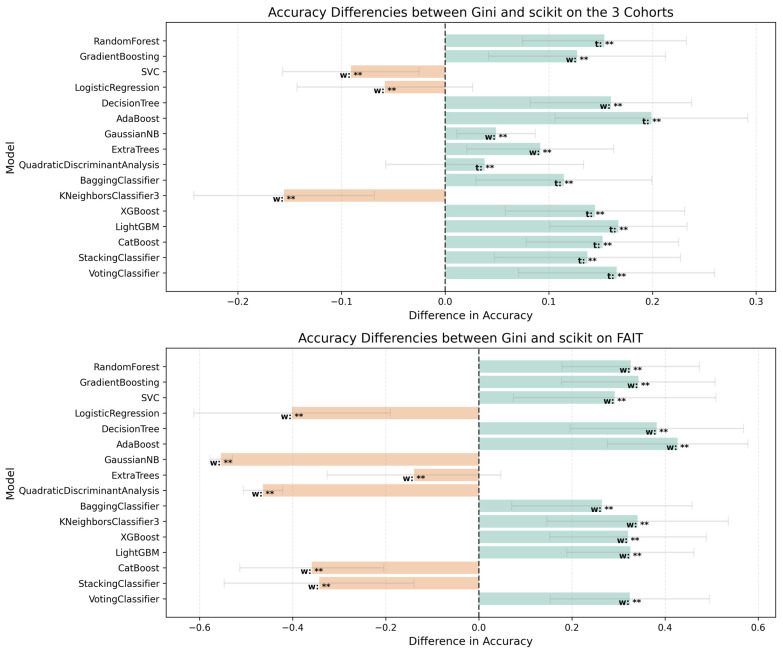
Accuracy differences between Gini and F one-way ANOVA FS methods across datasets. The plots show the differences in model accuracy for ML models trained using features selected by the Gini index and F one-way ANOVA test methods. (**Top**) Results for the combined Cohort 1 and 2 dataset. (**Bottom**) Results for the Cohort 3 (FAIT) validation dataset. Positive differences (light teal) indicate better performance with Gini-selected features, while negative differences (light coral) indicate better performance with F one-way ANOVA features. Error bars represent the combined standard deviations of the two FS methods. Statistically significant differences are marked with asterisks, ** (*p* < 0.01), with t and w denoting the test type (paired t-test or Wilcoxon Signed-Rank test, respectively). Dashed vertical lines at x = 0 indicate no difference in performance. Positive differences (light teal) indicate better performance with Gini-selected features, while negative differences (light coral).

**Figure 6:**
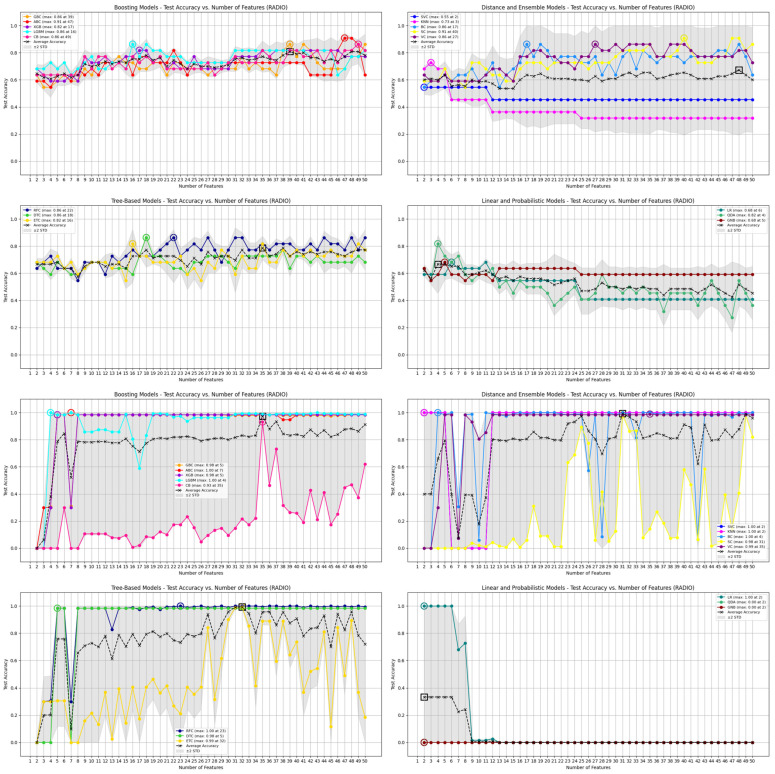
Top section: Results of 16 ML models, trained using features selected by the GINI FS method, subdivided into four groups—Boosting Models, Tree-Based Models, Distance and Ensemble Models, and Linear and Probabilistic Models—on data from cohorts 1 and 2. Each plot shows the test accuracy of the models as a function of the number of selected features, highlighting performance trends within each group. Bottom section: Comparison of the same 16 ML models, also trained using features selected by the GINI FS method, on the Cohort 3 (FAIT) dataset.

**Figure 7:**
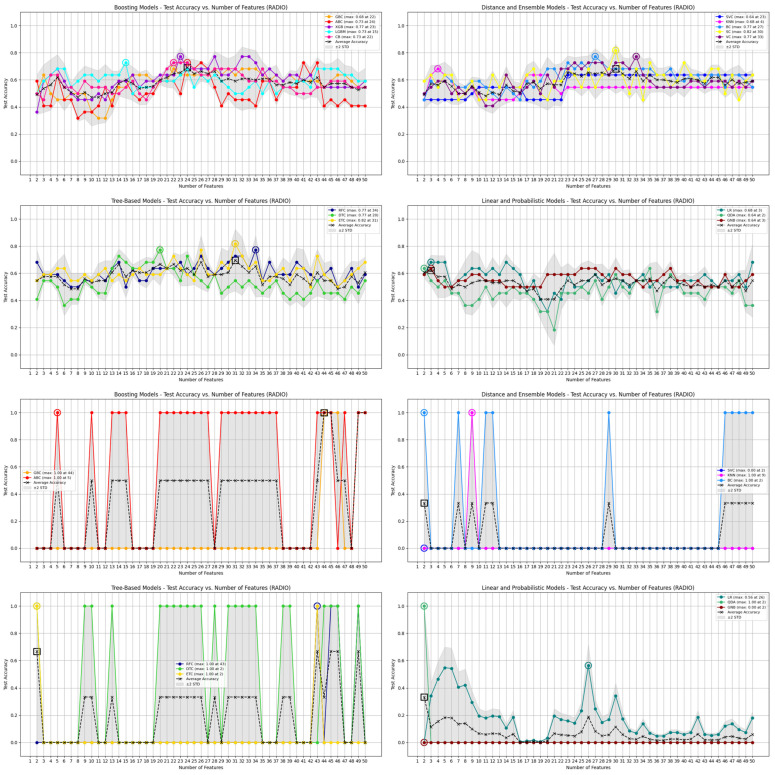
Top section: Results of 16 ML models, trained using features selected by Scikit-learn’s FS methods, subdivided into four groups—Boosting Models, Tree-Based Models, Distance and Ensemble Models, and Linear and Probabilistic Models—on data from cohorts 1, 2, and 3. Each plot shows the test accuracy of the models as a function of the number of selected features, highlighting performance trends within each group. Bottom section: Comparison of the same 16 ML models, also trained using features selected by Scikit-learn’s FS methods, on the FAIT dataset selected using Scikit-learn methods.

Table 2 compares the performance of the 16 ML models trained on radiomic features selected by either the Gini index or F one-way ANOVA, evaluated across both internal cross-validation (Cohorts 1 and 2) and external multi-center validation (Cohort 3). Each model’s performance is reported in terms of mean accuracy, standard deviation, and observed minimum and maximum values. Figure 5, Figure 6 and Figure 7 further visualize the accuracy distributions across feature subset sizes. While confidence intervals were not calculated for every configuration due to the large number of iterations, the summary metrics provide a reliable assessment of performance variability.

In internal cross-validation, Gini-selected features consistently yielded higher accuracy and greater stability. For example, the RFC and SC achieved a peak cross-validation accuracy of 90.9%, while models like KNN performed less reliably (mean: 38.4%). In contrast, models using F-statistic-selected features demonstrated overall lower mean accuracy, with top performers such as ETC and SC reaching 81.8%, but still falling short of the best Gini-based models. The multi-center validation results further highlight the robustness of Gini-selected features.

**Figure 8:**
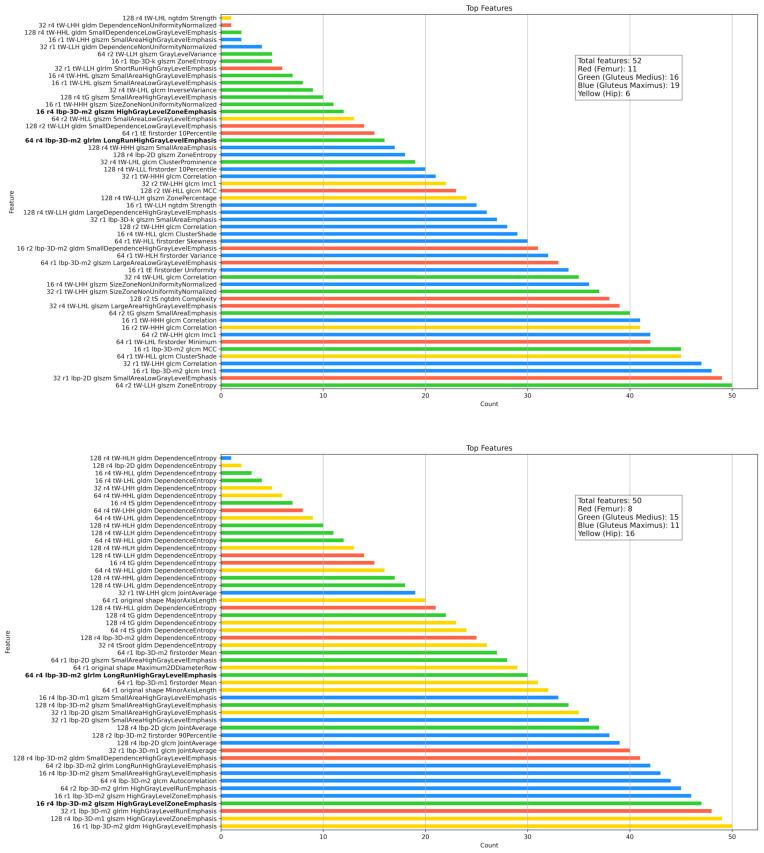
Top bar chart: Frequency and names of features selected by fine-tuning FS using the Gini index. The features are categorized by anatomical regions: femur (red), hip (yellow), gluteus medius (green), and gluteus maximus (blue). Features selected by both Gini and F-statistic (f classif) methods are highlighted in bold. A summary box indicates the total features and the counts for each region. Bottom bar chart: Frequency and names of features selected by fine-tuning FS using the F one-way ANOVA. The color scheme and bold formatting for overlapping features are consistent with the Gini selection chart.

Several Gini-trained models—RFC, GBC, and ABC—achieved mean accuracies above 90%, and 13 out of 16 models reached perfect accuracy (100%) when classifying symptomatic cases from the FAIT dataset. Meanwhile, F-statistic-based models like GNB and QDA struggled significantly, with accuracy dropping to 0% in some cases. A Mann–Whitney U test comparing the accuracy distributions across all the models confirmed a significant advantage for Gini-based selection, both internally (U = 461,066.5, *p* = 3.09 × 10^−53^) and externally (U = 395,042.0, *p* = 3.52 × 10^−16^). Notably, RFC, XGB, LGBM, and VC showed significantly better accuracy using Gini-selected features in both internal and external settings (*p* < 0.01), while only SVC and QDA slightly favored F-statistic-based features. Despite some F-statistic-trained models reaching perfect classification on Cohort 3, their overall performance was unstable—fluctuating between 0% and 100% across feature subsets—raising concerns about reproducibility and clinical applicability. Figure 8 further compares the anatomical distribution of features selected by both FS methods. Across the 50 feature subset range (P(50,1) = 1275 combinations), the Gini index consistently prioritized a core group of 52 dominant features, primarily from the gluteus maximus (19 features), gluteus medius (16), femur (11), and hip (6). In contrast, F-statistic-based selection emphasized the hip (16 features) and femur, with a more dispersed pattern across regions. These differences reflect the distinct selection logics of the methods—Gini favors features that reduce impurity in decision trees, while F-statistic prioritizes group variance. Together, these findings suggest that Gini-based FS provides a more stable, interpretable, and anatomically informative framework for robust model development across both bone and soft-tissue regions.

To complement the accuracy results description, Table 3 summarizes each model’s performance using the best feature selection strategy (Gini). For each classifier, we report the cross-validation accuracy on the internal dataset, the accuracy (detection rate) on the multi-center validation cohort (Cohort 3), the number of features used, and a composite performance score. This score ranks models based on a weighted combination of generalizability, internal robustness, and model simplicity, and is computed using the formula:

$$Score=\frac{{ACC({NF}_{OPT})}_{MCV}}{100}+\frac{{ACC({NF}_{OPT})}_{CV}}{100}+\frac{1}{2}\left(1-\frac{{NF}_{OPT}}{50}\right)$$

The resulting ranking provides a comprehensive evaluation of classifier performance by considering both predictive strength and feature efficiency. The highest-ranking models were LGBM, BC, and DTC. These models showed strong accuracy on both internal and external datasets while using a relatively small number of features. For example, LGBM achieved 100% accuracy on the cross-validation set and 86% for the multi-center cohort, and used only 16 features, indicating a highly effective and compact configuration. Other models, such as RFC, XGB, and VC, also performed well. These models maintained consistent accuracy across internal and external datasets while operating with moderate feature complexity. Their strong generalizability highlights the suitability of tree-based ensemble methods for radiomic classification tasks. Some models demonstrated signs of overfitting or limited external robustness. The SC reached 91% accuracy in the cross-validation but only 58% in the external testing, suggesting that it may have overfitted to the single-center training data. QDA and GNB performed moderately on the internal dataset but failed on the external validation, with 0% detection accuracy. This result indicates that their statistical assumptions, including the Gaussianity of the feature distribution, may not hold for real-world, heterogeneous MRI data. The behavior of the classifiers aligned with their algorithmic characteristics. Boosting models, including LGBM, ABC, and GB, captured complex interactions between features and performed reliably across datasets. Tree-based models, such as RFC and DT, were similarly robust, with stable accuracy and interpretable decision logic. In contrast, linear models like LR and probabilistic models such as QDA and GNB performed less reliably, especially when applied to external data. These models may be more sensitive to variability in feature distribution. Distance-based models, such as KNN and SVC, showed moderate internal performance but were less stable in external validation, likely due to sensitivity to high-dimensional feature spaces.

## 4. Discussion

Radiomics-based approaches have demonstrated significant potential in aiding the diagnosis of FAI from MRI, offering a quantitative, reproducible alternative to traditional image-based methods reliant on subjective evaluations. Previous studies have shown that radiomics, combined with ML, can effectively differentiate FAI subtypes, achieving 97% accuracy in distinguishing S from A joints [16] and 100% accuracy in separating H from S joints using RFC models with optimized features [15].

However, prior studies have typically focused on a single classifier, used small or augmented datasets, and lacked external validation. To our knowledge, this is the first study to systematically evaluate multiple machine learning models trained on radiomic features for FAI, using a single-center dataset and testing generalizability across a large, diverse multi-center validation cohort. This framework directly mirrors the clinical deployment pipeline—developing models locally and testing them globally—and demonstrates that radiomics can be both scalable and reliable in unseen clinical contexts [34].

Our study builds on these findings, achieving up to 90.9% accuracy in classifying hip joints as H, S, and A in Cohorts 1 and 2, and reaching 100% accuracy for the detection of FAI in Cohort 3. It is important to note that the external validation cohort (Cohort 3) included only symptomatic (S) cases. Therefore, reported performance on this cohort reflects the detection rate, or true positive rate, for symptomatic FAI, rather than multi-class accuracy.

While a previous study by Montin et al. [15] focused on single-center datasets with limited patient diversity, this study trained models on a single-center dataset comprising 41 patients (82 hip joints) and validated them on a multi-center one of 185 patients, significantly enhancing the generalizability and robustness of the produced models. The RFC model in this study maintained strong performance across diverse datasets, achieving up to 86.4% accuracy in Cohorts 1 and 2, and replicating Montin et al.’s perfect classification of H vs. S cases. These results validate the effectiveness of our feature selection strategy and highlight that radiomic features optimized on single-center data can generalize to multi-center datasets, without requiring site-specific adaptation.

Importantly, while our training data was acquired under a single standardized Dixon protocol, the external cohort reflects real-world heterogeneity. This includes variability in acquisition parameters such as TR, TE, contrast use, and scanner platform. The model’s ability to generalize under such variation suggests resilience to protocol differences, although future studies will formally quantify feature robustness under controlled multi-sequence imaging conditions.

These results were further supported by a score-based ranking of all the classifiers that integrated cross-validation accuracy, external validation performance, and feature parsimony. Models such as LGBM, BC, RFC, and DT achieved the highest scores. These classifiers demonstrated consistent generalization across institutions and required relatively few features, making them attractive for clinical use. In contrast, models like SC, QDA, and GNB, despite reasonable performance on internal datasets, showed poor or unstable results in external validation. This suggests potential overfitting or an inability to accommodate the heterogeneity inherent in multi-institutional data. This finding is particularly relevant to the challenge of clinical translation: in real-world scenarios, diagnostic tools are often developed using data from a single institution but expected to perform reliably across diverse clinical environments. Our results suggest that radiomics-based models, when coupled with carefully designed feature selection, maintain diagnostic accuracy even under such deployment conditions. Unlike KNN, which was used in Montin et al.’s study [15], and which exhibited declining performance with increased feature counts, the RFC consistently delivered high diagnostic precision, emphasizing its scalability and adaptability. FS is essential for developing accurate ML models in radiomics, in which high-dimensional features complicate training and interpretation. Selecting relevant radiomic features improves model interpretability, efficiency, and generalizability, facilitating the development of reproducible diagnostic tools with clinically meaningful predictors [35]. In this study, we implemented an FS pipeline that included a standardized four-step process, followed by refinement using the Gini index and F one-way ANOVA for feature optimization. The Gini index measures a feature’s impact on reducing node impurity in decision trees [28], identifying strong feature subsets for tree-based classifiers. In contrast, F one-way ANOVA ranks features using statistical tests like F-statistics [36], suitable for datasets with distinct groups. Gini-selected features outperformed the F one-way ANOVA, particularly with ensemble methods such as RFC and gradient boosting, achieving over 90% accuracy on Cohorts 1 and 2. We deliberately excluded dimensionality reduction techniques such as PCA, UMAP, and t-SNE, as these methods generate combinations of features that obscure their anatomical origins. In contrast, our approach retained 1183 individually traceable features, allowing for explainable ranking and clearer interpretation in clinical contexts. Focusing on interpretable FS supports creating diagnostic models that clarify FAI pathophysiology rather than just improving classification metrics. This design choice supports clinical adoption by ensuring that selected features are not only statistically robust but also anatomically and pathophysiologically interpretable. The dataset’s size and diversity eliminated the need for data augmentation through image transformation [37], ensuring robust model performance.

Previous radiomics studies for FAI classification focused on the acetabulum and femur, using the textural features derived from GLCM and GLRLM, histogram-based features and shape descriptors. Our study expands to additional regions like the gluteus medius and maximus and employs advanced image transformations, enhancing the detection of subtle texture variations. This shift in FS shows a move from global intensity measures like histogram skewness and quantiles to localized texture features. While earlier studies emphasized anterior–posterior and superior–inferior shape variability [16], these now play a smaller role. New descriptors, such as region-specific skewness in the gluteal areas, offer a detailed musculoskeletal analysis. By including the gluteal musculature beyond the acetabulum–femur focus, we provide a more complete examination of FAI.

Beyond joint morphology, our findings emphasize the role of periarticular muscle remodeling in FAI pathophysiology. Prior studies have reported asymmetrical cross-sectional areas and altered activation patterns in the gluteus maximus and medius of symptomatic patients, often correlating with pain and functional limitation [11]. These neuromuscular adaptations may reflect compensatory mechanisms and are detectable through texture features. By incorporating radiomic descriptors from these muscles, our models extend beyond static shape analysis toward a more functional and physiologically-informed classification of FAI. External validation is crucial for ML models, especially in clinical settings [38]. This study validated models on the Cohort 3 dataset of nearly 200 cases from seven centers with varied scanners (1.5T and 3T), imaging protocols, and demographics, testing their adaptability. Remarkably, the models trained on the smaller datasets of Cohort 1 and 2 maintained their accuracy when applied to the symptomatic FAI cases in Cohort 3, yielding perfect results. This level of performance across a heterogeneous, multi-institutional cohort strengthens the case for clinical deployment of radiomics-based diagnostic models and demonstrates that these features are not overly dependent on specific scanner settings, institutions, or imaging protocols. Avoiding Type I (false positive) and Type II (false negative) errors is essential to prevent unnecessary actions or delayed treatments. The high diagnostic accuracy in a large, diverse cohort shows that the identified radiomic features are genuinely predictive, not just tailored to the small sample used for training. We believe that the observed generalizability is due to the feature selection process being focused on statistical significance and clinical relevance. By integrating methods to identify informative features, the resulting subset withstands different imaging protocols, scanners, and inter-patients variability, ensuring predictive signatures are useful beyond controlled settings and supporting precision medicine and value-based healthcare goals [39].

To enable real-world clinical translation, prediction outputs should include mechanisms to manage false positives and false negatives. False positives could be routed for secondary radiologist review or additional imaging (e.g., MR arthrography or dynamic CT), while false negatives might be reduced through model threshold tuning based on the clinical context (e.g., screening vs. surgical planning). Incorporating explainability tools such as feature importance heatmaps could also guide clinical interpretation and override decisions when appropriate.

To complement this multicenter validation and further assess clinical applicability, a prospective, real-world deployment study is currently being planned. This follow-up will involve integrating the trained pipeline into institutional PACS systems and evaluating model performance in routine clinical workflows across multiple hospital sites, including several FAIT-affiliated centers. The goal of this prospective phase is to validate both prediction accuracy and usability in operational conditions, while also assessing interpretability and clinical trustworthiness.

While our study has several strengths—including standardized radiomics extraction, comparative evaluation of 16 classifiers, and robust external validation—some limitations remain. Our training cohort was relatively small, and subtype-level annotations (e.g., cam, pincer) were not available. However, our findings remain clinically meaningful as they reflect the real-world challenge of classifying symptomatic presentations, independent of morphological subtype. The models’ ability to generalize across scanner types and institution settings supports their relevance for future prospective trials and eventual integration into diagnostic workflows.

Overall, this study offers an important step toward clinical translation by demonstrating that radiomics pipelines trained on single-center MRI data can achieve perfect generalization to unseen, multi-center cohorts. While full clinical integration will require prospective validation and workflow integration, our findings suggest that the foundational tools are in place. This positions radiomics not only as a research method but as a viable solution for real-world, multi-institutional diagnostics in musculoskeletal imaging.

## 5. Limitations

Despite the promising results, this study has several limitations. First, although MRI is the gold standard for evaluating FAI, our pipeline relies exclusively on a single imaging modality, which may not fully capture dynamic joint behavior or functional impairments. Second, while external validation was conducted on a multi-center cohort, the training dataset was limited to a relatively small number of cases from a single institution. This limitation was partially mitigated through rigorous cross-validation and by demonstrating strong generalizability to diverse external data. Third, the absence of explicit cam, pincer, or mixed subtype labels in our dataset prevented subtype-specific performance evaluation. Although all the segmentations were visually reviewed and refined using the STAPLE algorithm to enhance robustness, we did not perform a quantitative comparison with manual segmentations. Future work will include a segmentation sensitivity analysis based on Dice similarity and Hausdorff distance metrics, and may explore alternative ensemble-based fusion strategies. In addition, demographic and clinical variables such as age, sex, BMI, and physical activity level were not consistently available across the retrospective dataset, preventing subgroup performance analysis. We plan to address this in future prospective studies through covariate-adjusted modeling to better understand model behavior across diverse populations.

Finally, although we evaluated two complementary feature selection strategies (Gini index and F-statistic), future work could explore deep feature learning or hybrid methods to further optimize model performance.

## 6. Conclusions

This study presents a fully automated radiomics and machine learning pipeline for diagnosing femoroacetabular impingement, encompassing segmentation, multi-region feature extraction, and classification. Compared to prior work, our approach expands the anatomical scope beyond joint morphology to include gluteal musculature, and evaluates a wide range of classifiers across a structured feature selection framework. By training on single-center data and externally validating on a heterogeneous, multi-center cohort, we demonstrated that radiomic features can generalize across scanners, protocols, and institutions without retraining—underscoring their potential for clinical translation

These findings support the growing emphasis on standardized, automated diagnostic tools for musculoskeletal imaging [40]. Our pipeline offers a reproducible and interpretable approach that may enhance diagnostic consistency, reduce reliance on subjective evaluation, and facilitate earlier, more personalized treatment planning for FAI.

In addition to validating generalizability, we introduced a composite scoring framework to evaluate classifiers based on accuracy, external robustness, and model compactness. This analysis revealed that LGBM, BC, RFC, and DT offered the best trade-offs between performance and simplicity. These models demonstrated consistent accuracy across internal and multi-center datasets while using a relatively small number of features. Their robustness and efficiency make them particularly well suited for clinical deployment, where interpretability and generalizability are essential.

Looking forward, this framework can be extended to additional patient populations, integrated into prospective trials, and deployed in scalable platforms such as cloud-based web applications [41]. Beyond FAI, our modular architecture provides a generalizable template for applying radiomics and AI to other joint and soft-tissue conditions in orthopedic and sports medicine.

## Figures and Tables

**Table 1:** A summary of cohort characteristics, including FAI subtypes where applicable.

Cohort	Description	Population	FAI Labels	Phenotype Information
Cohort 1	Bilateral hip MRI scans of unilateral FAI	22 F, 36 ± 8 yrs; 20/31 w/contrast	S, A	All classified as mixed cam + pincer
Cohort 2	Bilateral MRI scans of healthy volunteers	5 F, 32 ± 6 yrs; no contrast	H	None
Cohort 3	Unilateral scans from 7-center FAIT study	123 F, 36.3 ± 10 yrs; mixed contrast	S	140 cam, 28 cam + dysplasia, 2 pincer, 14 cam + pincer, 1 dysplasia

**Table 2:** Mean, (Standard Deviation), and [Minimum, and Maximum] test accuracy for each ML model trained using features selected by Gini index and from the F one-way ANOVA. The Cross validation column refers to the accuracy of the testing on Cohorts 1 and 2, while the Multi-Center Validation refers to the results of the models tested on data from Cohort 3.

ML Models	Gini FS		F one-Way ANOVA	
	Cross-Validation	Multi-Center Validation	Cross-Validation	Multi-Center Validation
RFC	75.5 (7.7) [54.5–86.4]	92.8 (21.4) [0–100]	60.2 (8.1) [27.3–77.3]	10 (30.3) [0–100]
GBC	70.1 (7.2) [54.5–86.4]	91.6 (23.1) [0–100]	57.4 (9.8) [27.3–68.2]	12 (32.8) [0–100]
SVC	47.7 (4.7) [45.5–68.2]	86 (35.1) [0–100]	56.8 (8.4) [36.4–63.6]	0 (0) [0–0]
LR	49.3 (9.3) [40.9–68.2]	15 (34.6) [0–100]	55.1 (7.6) [31.8–68.2]	18.7 (18.7) [0–100]
DTC	68.4 (5.3) [59.1–86.4]	90.6 (27) [0–100]	52.4 (10.3) [27.3–77.3]	48 (50.5) [0–100]
ABC	70.8 (7.6) [54.5–90.9]	93.6 (19.1) [0–100]	50.9 (11) [31.8–72.7]	56 (50.1) [0–100]
GNB	60.3 (2.7) [54.5–68.2]	0 (0) [0–0]	55.4 (4.8) [45.5–63.6]	0 (0) [0–0]
ETC	69.5 (6.4) [54.5–81.8]	46.4 (29.5) [0–100]	60.4 (7.8) [31.8–81.8]	6 (24) [0–100]
QDA	50.2 (10.6) [27.3–81.8]	0 (0) [0–0]	46.4 (8.5) [18.2–63.6]	2 (14.1) [0–100]
BC	72.2 (8.4) [54.5–86.4]	87.2 (30.3) [0–100]	60.7 (8.6) [31.8–77.3]	22 (41.8) [0–100]
KNN	38.4 (11.3) [31.8–72.7]	88 (32.8) [0–100]	53.9 (6.2) [31.8–68.2]	2 (14.1) [0–100]
XGB	74.2 (7) [59.1–81.8]	91.8 (23.3) [0–100]	59.7 (10.3) [22.7–77.3]	52 (50.5) [0–100]
LGBM	75.8 (6.2) [63.6–86.4]	91.7 (20.3) [0–100]	59.1 (7.1) [31.8–72.7]	34 (47.9) [0–100]
CB	72.9 (6.3) [63.6–86.4]	21.8 (22.5) [0–100]	57.7 (8.4) [27.3–72.7]	2 (14.1) [0–100]
SC	72.9 (9.1) [54.5–90.9]	24.9 (31.9) [0–98.4]	59.2 (8.9) [36.4–81.8]	4 (19.8) [0–100]
VC	74.7 (9.4) [59.1–86.4]	90.6 (24.7) [0–100]	58.2 (9.5) [27.3–77.3]	12 (32.8) [0–100]

**Table 3:** Ranked summary of model performance using Gini-based feature selection. For each machine learning classifier, we report the accuracy (detection rate) on the external multi-center validation cohort (Cohort 3), the cross-validation accuracy (Cohorts 1 and 2) on the internal dataset, the number of features used by the model, and a composite performance score. The multi-center validation accuracy value is calculated by applying the model trained on the number of features that yielded the highest cross-validation performance. Models are ranked in descending order by score, with higher values indicating better trade-offs between performance and simplicity.

Ml Models	Multi-Center Validation	Cross Validation	Number of Features	Score
LGBM	86%	100%	16	2.2
BC	100%	86%	17	2.19
DTC	98%	86%	18	2.16
XGB	99%	82%	17	2.14
RFC	99%	86%	22	2.13
LR	100%	68%	6	2.12
VC	98%	86%	27	2.07
SVC	100%	55%	2	2.03
GBC	99%	86%	39	1.96
ABC	99%	91%	47	1.93
KNN	100%	73%	33	1.9
CB	86%	99%	49	1.86
SC	58%	91%	40	1.59
ETC	40%	82%	16	1.56
QDA	0%	82%	4	1.28
GNB	0%	68%	5	1.13

## Data Availability

The data presented in this study are available on request from the corresponding author.
